# OsCBL1 Modulates the Nitrate-Induced Phosphate Response by Altering OsNLP4 Cytoplasmic-Nucleus Shuttling

**DOI:** 10.1186/s12284-025-00768-6

**Published:** 2025-03-10

**Authors:** Zhao Hu, Yunting Tang, Suping Ying, Jiawei Niu, Ting Wang, Huaiyi Zhu, Xiaojue Peng

**Affiliations:** 1 College of Life Science, Nanchang University, Nanchang, 330031 China; 2 Department of Chemistry, University of Kentucky, Lexington, KY40506 USA; 3 College of Biological Sciences and Technology, Taiyuan Normal University, 030619 Taiyuan, China

**Keywords:** *OsCBL1*, *OsNLP4*, *OsNRT1.1B*, *OsSPX4*, Nitrate-induced phosphate signaling, Cytoplasmic-nucleus shuttling

## Abstract

Nitrate can directly activate phosphate (Pi) starvation signaling, ultimately promoting plant growth by enhancing phosphorus absorption and utilization and optimizing the balance of nitrogen and phosphorus nutrients. However, the complex mechanisms by which plants integrate complex nutrient signals from nitrogen to phosphorus are not well understood. This study highlights the importance of Calcineurin B-like protein-1 (OsCBL1), a calcium sensor, in coordinating nitrogen and phosphorus signaling in rice. Knockdown of *OsCBL1* in rice reduced the expression of genes involved in nitrate-induced Pi starvation responses. In high nitrate conditions, *OsCBL1*-KD plants displayed diminished biomass gain, unlike the wild-type rice, which thrived under elevated phosphate levels. In *OsCBL1*-KD plants, OsSPX4, a key repressor in nitrogen and phosphorus signaling, remains undegraded in the presence of nitrate due to the significantly reduced expression of *OsNRT1.1B*. Moreover, the *OsCBL1* knockdown hampers the movement of the nitrogen-related transcription factor, OsNLP4, from the cytoplasm to the nucleus when nitrate is present. This impedes the expression of *OsNRT1.1B*, as OsNLP4 can directly bind to the promoter of *OsNRT1.1B* nitrate responsive cis-element (NRE) and activate its expression. In summary, these findings suggest that *OsCBL1* plays a pivotal role in regulating *OsNRT1.1B* expression by managing the transport of OsNLP4 between cytoplasm and nucleus in response to nitrate availability. This regulation subsequently influences the phosphate response triggered by nitrate and optimizes the coordinated utilization of nitrogen and phosphorus.

**Supplementary Information:**

The online version contains supplementary material available at 10.1186/s12284-025-00768-6.

## Introduction

Nutrient balance is crucial for healthy plant growth and productivity, with the appropriate proportion of each necessary nutrient at each growth stage being essential. Plants have developed mechanisms to sense and integrate nutrient signals to coordinate absorption and utilization, aiming to avoid over-or under-nourished conditions and optimize nutrient use (Cahill et al. 2010; Poza-Carrión and Paz-Ares 2019). Nitrogen (N) and phosphorus (P) are key nutrients for plant growth and development, with a suitable N: P supply significantly enhancing plant growth and production (Marklein and Houlton 2012; Ye et al. 2019). Earlier studies indicated that increased N supply can promote the transcription of phosphate transporter genes, which in turn promotes P uptake and accumulation (Deng et al. 2016; Feng et al. 2017; Lu and Tian 2017). However, the molecular mechanism by which plants integrate N and P signaling to achieve an optimal N-P nutrient balance remains largely unknown.

Nitrate functions not only as a primary N source but also acts as a vital signaling molecule that influences gene expression related to various developmental processes and triggers nutritional responses in multiple metabolic pathways(Crawford 1995; Vidal et al. 2020). Recent years have seen the discovery of numerous key factors involved in nitrate signaling and response. For example, in *Arabidopsis*, AtNRT1.1/CHL1 functions as both a nitrate transporter and sensor, identifying changes in nitrate concentration via a Thr101 phosphorylation switch (Ho et al. 2009). In rice, OsNRT1.1B, a homologous gene of AtNRT1.1, also functions as a nitrate sensor, facilitating nitrate signaling transduction (Hu et al. 2015). The NIN-LIKE PROTEIN family (NLPs) of transcription factors regulate nitrogen signaling and assimilation by binding to nitrate-responsive cis-elements and activating related gene expression (Konishi and Yanagisawa 2013, 2014). Among the NLPs, AtNLP7 has been identified as a key transcription factor for nitrate response and influences the movement between cytoplasm and nucleus, as well as the expression of nitrate-induced genes (Marchive et al. 2013; Liu et al. 2017). The enrichment of AtNLP7 in the nucleus in response to nitrate is initiated by phosphorylation from Ca^2+^ sensor protein kinases (CPKs) and is dependent on the Ca^2+^ signal pathway (Liu et al. 2017). In rice, OsNLP4, a homologous gene of AtNLP7, can translocate from the cytoplasm to the nucleus in response to nitrate and directly modulates the expression of genes involved in N uptake, assimilation, and signaling by binding to the nitrate responsive cis-element (NRE) region (Wu et al. 2021). However, it remains unclear whether this process in rice is also dependent on the Ca^2+^ signaling pathway in rice.

Phosphate, an essential nutrient for plants, also acts as a signaling molecule for gene expression and nutritional response (López-Arredondo et al. 2014). OsPHR2 is crucial for the up-regulation of the expression of PSI genes (Zhou et al. 2008). In contrast, OsSPX4 acts as a repressor of PSI gene expression. In sufficient- phosphate conditions, OsSPX4 protein senses high phosphate concentration and directly interacts with OsPHR2 to inhibit its translocation to the nucleus (Lv et al. 2014). Conversely, under low phosphate conditions, OsSPX4 degrades via 26 S proteasome, allowing OsPHR2 to enter the nucleus and activate PSI gene expression (Ruan et al. 2019). Other SPX proteins, such as OsSPX1, OsSPX2, and OsSPX6, can also inhibit the function of OsPHR2 by impeding its binding to target genes or its nucleus translocation (Wang et al. 2014; Zhong et al. 2018). These findings underscore the importance of SPXs-PHR2 module in the plant’s phosphate signaling.

Recently researches have highlighted the interplay between nitrate and phosphate signaling. Maeda et al. found that in *Arabidopsis*, *AtNIGT1* is regulated by both AtPHR1 and AtNLP, participating in two transcriptional cascades that establish a direct connection between phosphorus and nitrogen nutritional regulation (Maeda et al. 2018). Furthermore, AtNIGT1 proteins can suppress SPX gene expression by directly binding to SPX promoters, activating PHR in response to phosphate starvation signaling (Ueda et al. 2020). Moreover, AtNIGT1.1 and AtNIGT1.2 serve a dual role as activators of phosphate transporters and repressors of nitrate transporters, maintaining a balance between N and P uptake under conditions of limited phosphate and sufficient nitrate (Wang et al. 2020). Similarly, the OsNRT1.1B-OsSPX4 module in rice plays a crucial role in N and P signaling. OsNRT1.1B promotes the degradation of OsSPX4 via the 26 S proteasome in the presence of nitrate, releasing key transcription factors OsPHR2 and OsNLP3, and facilitating coordinated N and P signaling and utilization (Hu et al. 2019). These reports indicate that the OsNRT1.1B-OsSPX4 module is essential for regulating the plant response to nitrate and phosphate signaling. However, the molecular mechanism by which plants sense and transmit N and P signals upstream of the OsNRT1.1B-OsSPX4 module remains unclear. Our previous research demonstrated the involvement of the Ca^2+^ sensor protein OsCBL1 in N and P signaling, influencing seedling growth, yet their molecular mechanisms are still unknown (Hu et al. 2021). In this study, reducing *OsCBL1* in rice decreased the expression of specific genes involved in the response to nitrate-induced Pi starvation, ultimately affecting the growth enhancement provided by elevated phosphate levels under high nitrate conditions. The key repressor of N and P signaling, OsSPX4, remains stable in the presence of nitrate due to diminished expression of *OsNRT1.1B* in *OsCBL1* knockdown plants. Furthermore, the knockdown of *OsCBL1* impedes the translocation of OsNLP4, a nitrate-related transcription factor, from the cytoplasm to the nucleus in the presence of nitrate. Consequently, this results in a reduction in the expression of *OsNRT1.1B*, given that OsNLP4 has the capacity to bind directly to its promoter. In conclusion, our results indicate that *OsCBL1* regulates the expression of *OsNRT1.1B* by modulating the subcellular localization of OsNLP4 in response to nitrate availability, providing new insight into the coordination of N and P signaling pathways in plants.

## Materials and Methods

### Plant Materials and Growth Conditions

The *OsCBL1*-KD and WT plants used in this study have been reported in a previous study (Yang et al. 2015). The WT rice Shijin B and *OsCBL1*-KD plants in this study were used in hydroponic experiments following previously described methods (Hu et al. 2021, 2023). Seeds were germinated in a dark incubator at 30 ℃ for 2–3 days after surface sterilization with 5% NaClO. Seedlings were then transferred to an 8-L hydroponic box and grown in a growth chamber with a photoperiod of 12 h (light)-12 h (dark) (~ 200 µmolm^− 2^ s^− 1^) at 30 ℃/28 ℃ and 70% humidity for 30 days. The basal nutrient solution was described in the previous study (Hu et al. 2019). The basal nutrient solution contains macronutrients (in mM): (NH_4_)_2_SO_4_ (0.25), MgSO_4_·7H_2_O (0.54), CaCl_2_·_2_H_2_O (0.36), K_2_SO_4_ (0.1), KH_2_PO_4_ (0.18) and Na_2_SiO_3_·_9_H_2_O (1.6), and micronutrients (in µM): MnCl_2_·4H_2_O (9.14), H_3_BO_3_ (46.2), (NH_4_)_6_Mo_7_O_24_·4H_2_O (0.08), ZnSO_4_·7H_2_O (0.76), CuSO_4_·5H_2_O (0.32) and Fe (II)-EDTA (40), with the pH adjusted to 5.8. Low N and high N were supplied with 0.2 mM and 5 mM KNO_3_, respectively. Low P and high P were supplied with 0.018 mM and 0.18 mM KH_2_PO_4_, respectively. The nutrient solution was renewed every two days.

### Short-Term Nitrate Induction Assay

The short-term nitrate treatment protocol was implemented according to the method described previously (Hu et al. 2019). Initially, the seedlings were cultivated in a basal nutrient solution (0.25 mM (NH_4_)_2_SO4, 0.18 mM KH_2_PO_4_, and NO_3_^−^ free) for 3 weeks. Then, the seedlings were transferred to the basal nutrient solution containing 2.5 mM (NH_4_)_2_SO_4_ and 0.18 mM KH_2_PO_4_ under continuous light conditions for an additional 2 days. Finally, 5 mM KNO_3_ or 5 mM KCl was added to the same nutrient solution for 2 h. Roots of the seedlings were collected for gene expression analyses. The related primers are listed in Table S1.

### RNA Isolation and qPCR Analysis

RNA isolation and RT-qPCR analysis were conducted following previously published methods (Hu et al. 2021, 2023). Total RNA was isolated using TRNzol Universal (TIANGEN, Cat no. DP424), and reverse transcription was carried out with Fasting RT kit (TIANGEN, Cat no. KR116). qPCR was performed on a StepOnePlus Real-Time PCR system with Power SYBR Green Master Mix (Applied Biosystems). Data points were obtained from three biological replicates for each gene. Target gene expression was normalized using Actin1 as the housekeeping gene. The related primers are listed in Table S1.

### Subcellular Localization Assay in Rice Protoplasts

Rice protoplasts were isolated and transformed using established methods (Bart et al. 2006; Zhang et al. 2011). Rice protoplasts were prepared independently from the three groups of rice seedlings of the same line (WT or *OsCBL1*-KD-L11), resulting in three independent protoplasts for subsequent experiments. Protoplasts were efficiently extracted from 10- to 15-day-old rice shoots in an enzymatic digestion solution for 4 h at 28 °C. The resulting protoplasts were then carefully washed twice with W5 solution (154 mM NaCl, 125 mM CaCl_2_, 5 mM KCl, 0.18 mM KH_2_PO_4_, and 2 mM MES at pH 5.7) and then resuspended in MMG (0.4 M mannitol, 15 mM MgCl_2_ and 4 mM MES at pH 5.7) for successful transfection. The transfection solution, prepared by mixing plasmids, protoplasts, and PEG4000 solution in a volume ratio of 1:10:11, was incubated at room temperature for 30 min in the dark. The reaction was terminated by adding 500 µl of W5 solution.

The In-Fusion Cloning Kit (ClonExpress II One Step Cloning Kit, Vazyme, C122-01) was employed for the construction of all expression vectors. To analyze the stability of OsSPX4 in response to nitrate stimulation, we used the method described previously (Hu et al. 2019). The full-length CDS of *OsSPX4* without a stop codon was amplified from the cDNA of WT and cloned into the *HBT-eGFP* vector to generate *HBT-OsSPX4-eGFP*. The *HBT-OsSPX4-eGFP* plasmids were then transfected into WT and *OsCBL1*-KD rice protoplasts incubated in W5 solution for 12 h, respectively. The transfected protoplasts were centrifuged at 100 × g for 5 min and subsequently treated with W5 solution containing either 10 mM KNO_3_ or 10 mM KCl for 2 h. Fluorescence signals were then captured using a confocal laser-scanning microscope under the same threshold settings. The related primers are listed in Table S1.

To investigate the subcellular localization of OsNLP4 after induction by nitrate, the full-length CDS of *OsNLP4* without a stop codon was amplified from the cDNA of WT and cloned into the *HBT-eGFP* vector to generate *HBT-OsNLP4-eGFP*. The *HBT-OsNLP4-eGFP* vector was transfected into both WT and *OsCBL1*-KD rice protoplasts, which were treated with either 10 mM KNO_3_ or 10 mM KCl. Additionally, nuclear localization sequence (NLS)-mCherry was employed as a control for protoplast co-transfection and nuclear labeling (Liu et al. 2017; Huang et al. 2024). The fluorescence signals were captured using a confocal laser-scanning microscope. Relevant primer details are listed in Table S1.

### Luciferase Activity Assay in Rice Protoplasts

Rice protoplasts were isolated and transformed as described above. To assess the stability of OsSPX4 in the luciferase activity system, we followed the method described previously (Hu et al. 2019). The full-length sequence of *pUBI* was amplified from the modified binary vector plasmid *pCU* (*pCAMBIA1301-UBI*), as previously described (Chen et al. 2007). The full-length sequence of *pUBI* was cloned into the *pGreenII0800-LUC* (containing firefly luciferase (LUC) and pro35S-renilla luciferase (REN) vector for generating the *pGreenII0800-pUBI-LUC*. The full-length CDS of *OsSPX4* without a stop codon was amplified from cDNA of WT and cloned into the *pGreenII0800-pUBI-LUC*, resulting in the *pGreenII0800-pUBI-OsSPX4-LUC* vector. The *pGreenII0800-pUBI-OsSPX4-LUC* plasmid DNA was introduced into nitrate-free rice protoplasts isolated from both WT and *OsCBL1-KD* plants. After a 12-hour incubation in W5 solution, the transfected protoplasts were treated with 10 mM KNO_3_ or KCl for 2 h. Protoplast protein was extracted, and the REN and LUC activities were assessed using a dual-luciferase reporter assay system (Promega, E1910). This experiment was conducted with at least three biological replicates. Relevant primer details are listed in Table S1.

To analyze the transcriptional regulation of OsNLP4 on *OsNRT1.1B*, we followed the method described previously (Hu et al. 2023). The full-length CDS of *OsNLP4* was amplified from the cDNA of WT and cloned into the *pCAMBIA1301-UBI* vector for generating the effector (*pCAMBIA1301-UBI-OsNLP4*). The *OsNRT1.1B* promoter was amplified from the DNA of WT and cloned into the *pGreenII0800-LUC* fused with the firefly luciferase (LUC) gene to generate the reporter (*pGreenII0800-pNRT1.1B-LUC*). Co-transfection of *pCAMBIA1301-UBI-OsNLP4* and *pGreenII0800-pNRT1.1B-LUC* was performed, with the *pCAMBIA1301-UBI* vector without *OsNLP4* used as the negative control. REN was taken as a reference. Protoplast protein was extracted and used for the detection of REN and LUC activities using the Dual-Luciferase Reporter Assay System (Promega, E1910) after a 14 h incubation in W5 solution at room temperature in the dark. Relevant primer details are listed in Table S1.

To confirm the role of OsNRT1.1B in promoting OsSPX4 degradation, the full-length CDS of *OsNRT1.1B* was amplified from the cDNA of WT and cloned into the *pCAMBIA2300* vector for generating the *pCAMBIA2300-OsNRT1.1B*. Co-transfection of *pCAMBIA2300-OsNRT1.1B* plasmid (5–10 µg) with *pGreenII0800-pUBI-OsSPX4-LUC* into rice protoplasts was conducted, followed by a 12-hour incubation in W5 solution. The empty *pCAMBIA2300* vector without *OsNRT1.1B* was taken as the negative control. REN was used as a reference. Protoplast protein was extracted and used for the detection of REN and LUC activities using the Dual-Luciferase Reporter Assay System (Promega, E1910) after a 14-hour incubation in W5 solution at room temperature in the dark. Relevant primer details are listed in Table S1.

### Measurement of Nitrate Content

Nitrate content analyses were performed according to previously published methods (Cataldo et al. 1975; Yang et al. 2019). Briefly, fresh rice samples were collected and ground to powder in liquid nitrogen. Then 0.1 g of fresh tissue sample was suspended in 1 ml of water and incubated at 45 °C for 1 h. The supernatant and 5% (w/v) salicylic acid (1:4) were mixed in concentrated H_2_SO_4_ for 20 min. After the addition of 2 mL of 2 M NaOH, the solution was measured at 410 nm wavelength and the nitrate concentration was calculated from a standard curve.

### Data Analysis

Experimental data were collected to calculate averages and Standard Error of the Mean (SEM), with the number of biological replicates indicated in the legend of each figure. Statistical significance between the transgenic lines and WT plants was determined by Student’s t-test at *P* ≤ 0.05. All statistical analysis was performed using Prism 8 statistical software.

## Results

### Nitrate Can Promote Pi Utilization by Activating Phosphate Signaling

**Fig. 1:**
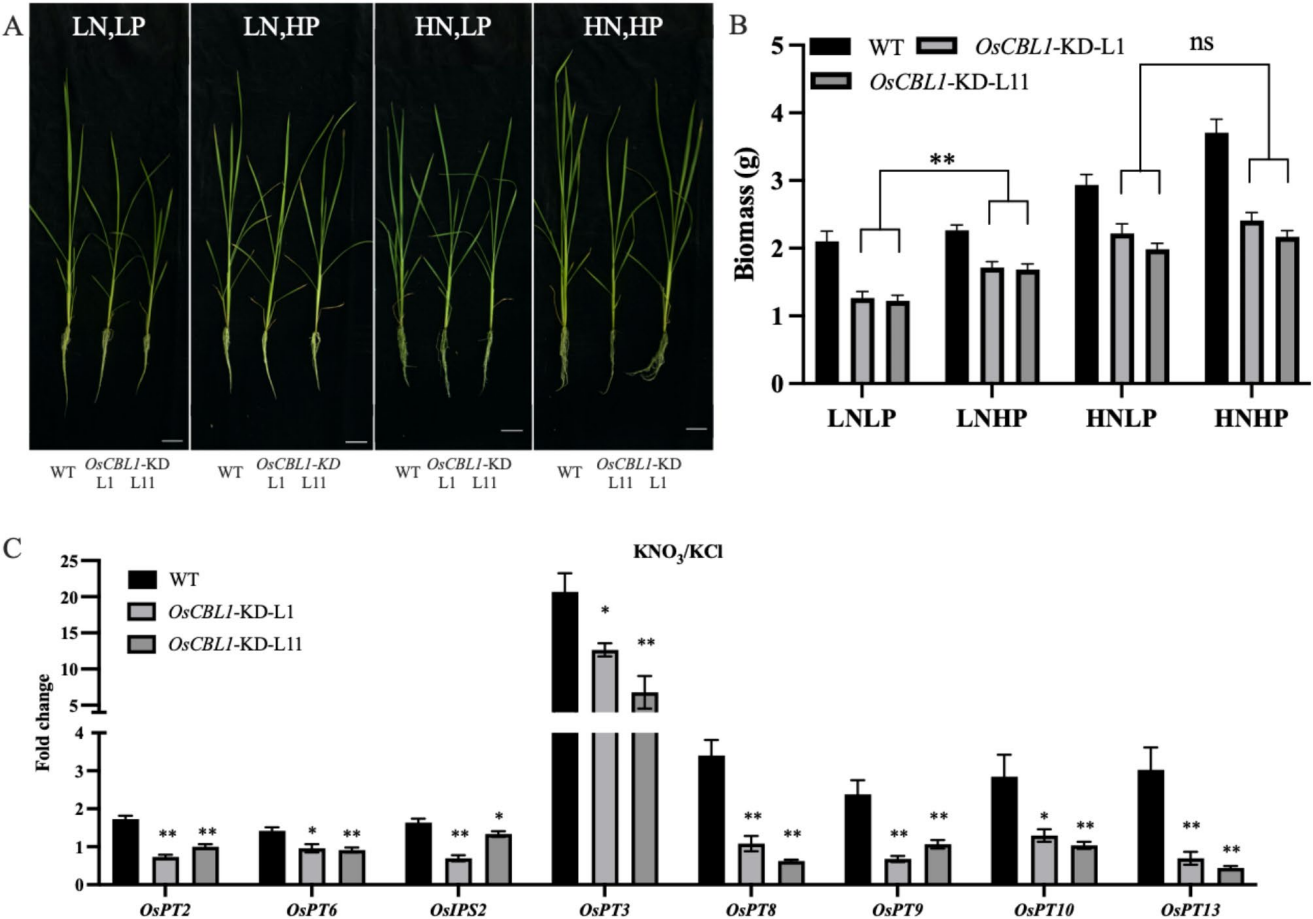
Nitrate-triggered phosphate signaling is depended on OsCBL1. **A** The growth phenotype of WT and *OsCBL1*-KD plants under varying N and P conditions. High nitrate (HN), 5 mM KNO_3_; low nitrate (LN), 0.2 mM KNO_3_; high phosphate (HP), 0.18 mM KH_2_PO_4_; low phosphate (LP), 0.018 mM KH_2_PO_4_. Scale bars = 5 cm. Images are representative of 9 rice plants. **B** The biomass of WT and *OsCBL1*-KD plants under varying N and P conditions. *n* = 9 biologically independent samples. The error bars represent ± SEM. ** *P* < 0.01 compared to the LP (*t*-test). **C** The expression of PSI genes in WT and *OsCBL1*-KD plants following treatment with KNO_3_ (5 mM). KCl treatment was used as the negative control. *n* = 3 biologically independent samples. The error bars represent ± SEM. * *P* < 0.05, and ** *P* < 0.01 compared to the WT (*t*-test)

A balanced supply of N and P nutrients can significantly enhance crop growth and yield (Luo et al. 2016). To investigate the relationship between N and P in plant growth, wild-type (WT) rice was cultivated under four different nitrate and phosphate supply conditions (high N high P, high N low P, low N high P, low N low P). Notably, growth and biomass increase in WT plants was only seen under high nitrate conditions with a high phosphate supply, while such an enhancement did not occur when nitrate was at low levels even with a high phosphate supply (Fig. 1A, B; Fig. S1A, S1B). This indicates that nitrate is essential for activating phosphate utilization. To further investigate this phenomenon, a short-term nitrate induction was conducted to determine if nitrate acts as a signaling molecule that stimulates phosphate-responsive gene expression. The short-term nitrate treatment resulted in the upregulation of several PSI genes, including *OsPT2*, *OsPT6*, *OsIPS2*, *OsPT3*, *OsPT8*, *OsPT9*, *OsPT10*, and *OsPT13*, compared to KCl treatment (Fig. 1C; Fig. S1C). These findings align with the research conducted by Hu et al. (2019), which indicated that nitrate can directly trigger genes related to phosphate starvation signaling, thus promoting plant growth.

### Knockdown of *OsCBL1* Impairs Nitrate-Induced Phosphate Signaling

Our previous study has indicated that *OsCBL1* regulates seedling growth by modulating N and P signaling (Hu et al. 2021). In this study, we first assessed the expression of *OsCBL1* under varying nitrate and phosphate supply conditions. The results showed that its expression was responsive to both nitrogen and phosphorus levels (Fig. S2). To further explore the molecular mechanism, we examined the transcription levels of nitrate-induced PSI genes in both WT and *OsCBL1*-KD plants. Compared to WT, knockdown of *OsCBL1* led to a significant reduction in the expression of nitrate-induced PSI genes (Fig. 1C), indicating the crucial role of OsCBL1 in nitrate-triggered phosphate signaling. In addition, we also cultivated *OsCBL1*-KD plants under varying nitrate and phosphate supply conditions (high N high P, high N low P, low N high P, low N low P). In all conditions, *OsCBL1*-KD plants gained lower biomass compared to WT (Fig. 1B). Interestingly, even under high nitrate conditions, the addition of extra phosphate did not result in a significant increase in biomass in *OsCBL1-KD* plants compared to those supplied with low phosphate levels (Fig. 1A and B). Conversely, WT plants showed a substantial biomass increase under high nitrate and high phosphate conditions (Fig. S1B). These results provided additional evidence for the pivotal role of *OsCBL1* in mediating plant responses to nitrate-induced phosphate signaling and maintaining a balance in N-P utilization. Collectively, these results highlight the reliance on nitrate-triggered phosphate signaling on OsCBL1.

### Knockdown of *OsCBL1* Impairs Nitrate-Triggered Degradation of OsSPX4

**Fig. 2:**
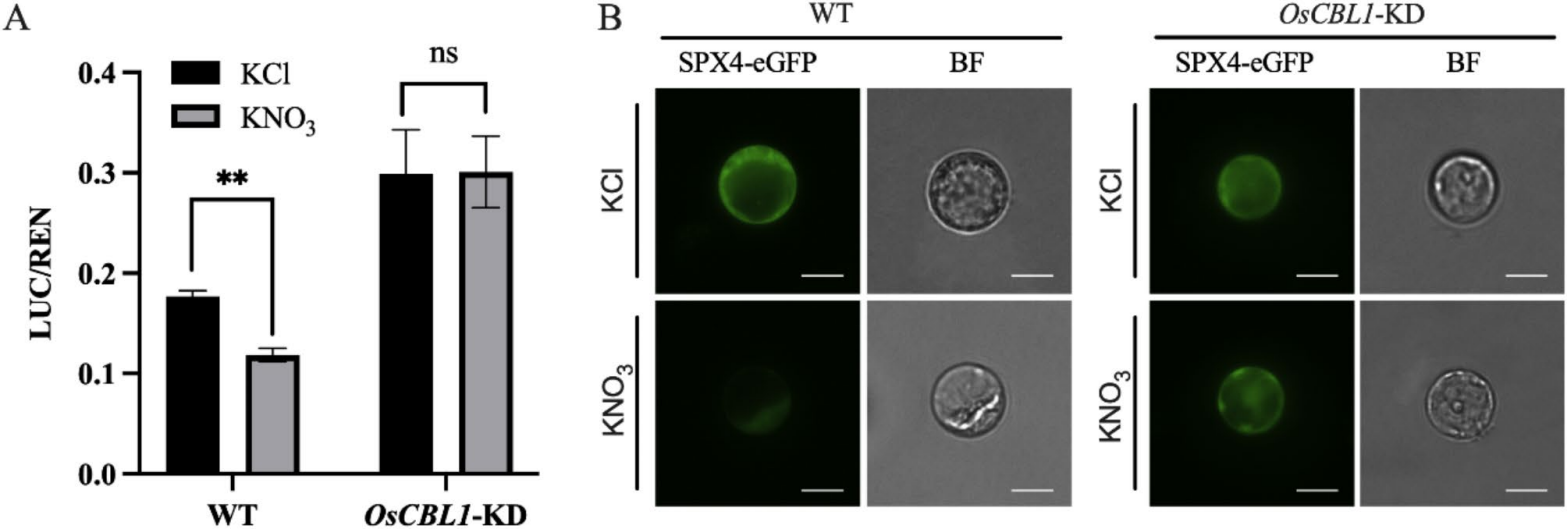
Nitrate-triggered OsSPX4 degradation is abolished in *OsCBL1*-KD protoplasts. **A** The comparative OsSPX4-fLUC activity in rice protoplasts of WT and *OsCBL1*-KD plants after treatment with KNO_3_ (10 mM). KCl treatment was used as the negative control. *n* = 3 biologically independent samples. The error bars represent ± SEM. ** *P* < 0.01 compared to the KCl (*t*-test). **B** The fluorescence of OsSPX4-eGFP in rice protoplasts of WT and *OsCBL1*-KD plants following treatment with KNO_3_ (10 mM). KCl treatment was used as the negative control. BF, bright filed. Scale bars, 5 μm

In a previous study, OsSPX4 was reported as a crucial repressor in nitrate-induced phosphate signaling. In addition, nitrate treatment was shown to enhance the degradation of the downstream protein OsSPX4 (Hu et al. 2019). To investigate the potential link between OsCBL1 and OsSPX4 in nitrate-induced phosphate signaling pathway, we conducted a luciferase activity assay to evaluate the stability of OsSPX4 protein both in WT and *OsCBL1*-KD rice protoplasts. As shown in Fig. 2A, the degradation of OsSPX4-firefly-luciferase (fLUC) in *OsCBL1*-KD plant protoplasts was significantly impeded compared to the wild type under nitrate-induced conditions. Furthermore, we also used eGFP-tagged OsSPX4 to observe its accumulation in rice protoplast. In WT rice protoplasts, exposure to nitrate resulted in a notable reduction in the fluorescence intensity of OsSPX4-eGFP. Conversely, no significant change was noted in the fluorescence of *OsCBL1*-KD protoplast between KCl treatment and KNO_3_ treatment (Fig. 2B, Fig. S3). Moreover, the expression of PSI genes was found to be reduced in *OsCBL1-KD* plants compared to the WT after KNO_3_ treatment (Fig. S4). These findings reinforce the idea that the nitrate-induced phosphate response depends on *OsCBL1*. In summary, these results demonstrated that knockdown of *OsCBL1* prevents the nitrate-induced degradation of OsSPX4, thereby influencing the phosphate signaling pathway triggered by nitrate.

### Knockdown of *OsCBL1* Reduces the Expression of *OsNRT1.1B*

**Fig. 3:**
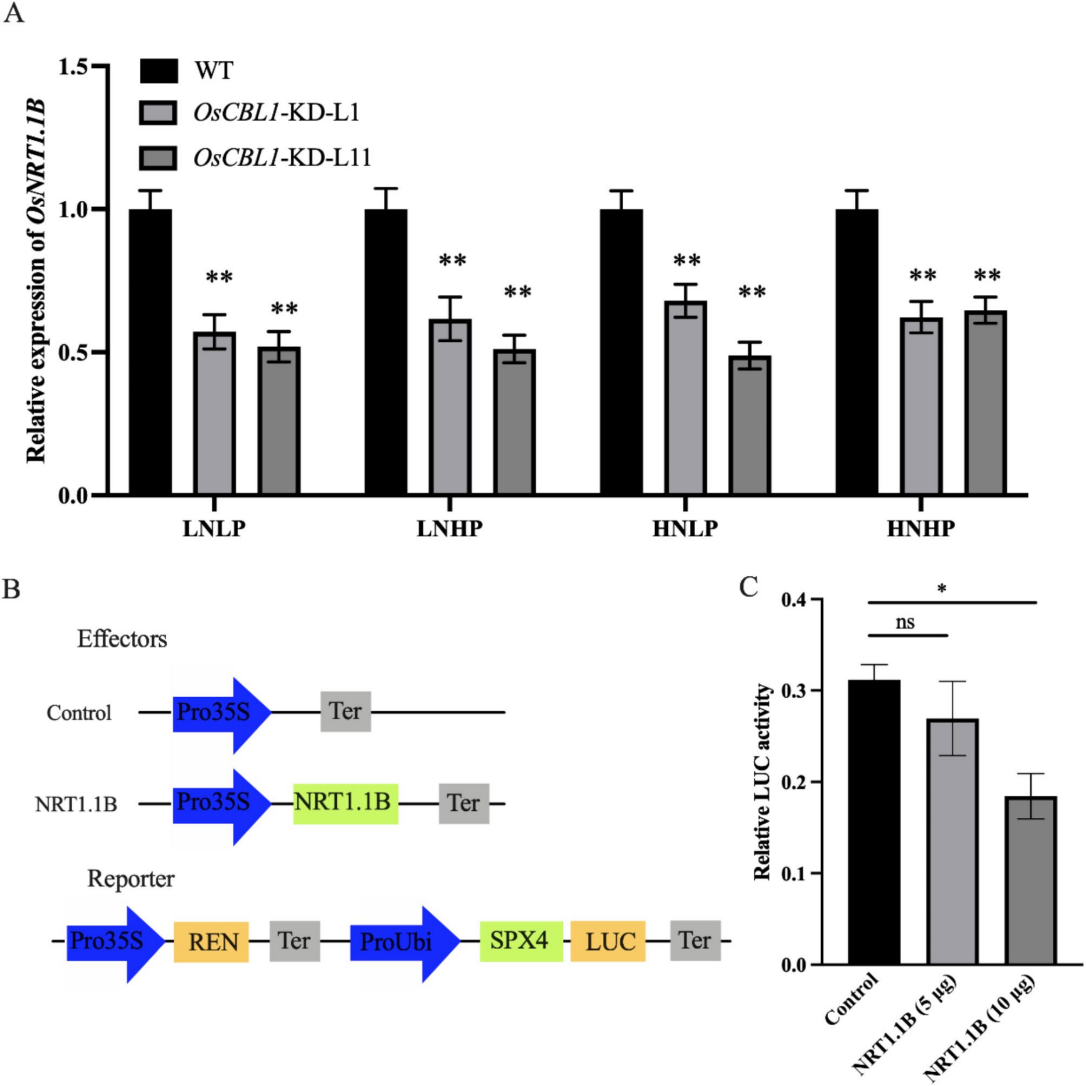
Knockdown of *OsCBL1* affects the transcription level of *OsNRT1.1B*. **A** The expression of *OsNRT1.1B* in the root of WT and *OsCBL1*-KD plants. *n* = 3 biologically independent samples. The error bars represent ± SEM. ** *P* < 0.01 compared to the WT (*t*-test). **B** Schematic illustration of the effector and reporter constructs. **C** Co-transformation of varied quantities of *Pro35S: NRT1.1B* with *ProUbi: SPX4-fLUC* in dual luciferase activity assays conducted in rice protoplasts. Numbers in brackets represent the amount of *Pro35S: NRT1.1B* construct co-transformed in each reaction. *n* = 3 biologically independent samples. The error bars represent ± SEM. * *P* < 0.05 compared to the control (*t*-test)

The results shown in Fig. 2 suggest that OsCBL1 plays a role in nitrate-induced phosphate signaling via OsSPX4-mediated pathway. However, Y2H assays indicated that OsCBL1 cannot directly interact with OsSPX4 (Fig. S5), suggesting that OsCBL1 does not directly activate the degradation of OsSPX4. Research by Hu et al., demonstrated that nitrate perception can enhance the OsNRT1.1B-OsSPX4 interaction, facilitating OsSPX4 degradation. In *nrt1.1b* mutants, this nitrate-induced degradation of OsSPX4 is inhibited (Hu et al. 2019). This prompted us to explore the regulatory link between OsNRT1.1B and OsSPX4 in *OsCBL1*-KD plants. We compared the transcription level of *OsNRT1.1B* in WT and *OsCBL1*-KD plants and found that the expression of *OsNRT1.1B* decreased in *OsCBL1*-KD plants under varying nitrate and phosphate supply conditions (Fig. 3A). This result indicates that *OsCBL1* functions upstream of *OsNRT1.1B* and regulates its expression. To assess the impact of decreased *OsNRT1.1B* transcript levels on OsSPX4 degradation, a modified luciferase activity assay was conducted in rice protoplasts using OsSPX4-fLUC as the reporter. The results showed a significant reduction in fluorescent signal with increasing amounts of *OsNRT1.1B* compared to the control, suggesting that reduced expression of *OsNRT1.1B* hampers OsSPX4 degradation (Fig. 3B and C). In summary, these results collectively demonstrate that *OsCBL1* is involved in nitrate-induced phosphate signaling, potentially mediated through the OsNRT1.1B-OsSPX4 module.

### OsCBL1 Regulates the Expression of *OsNRT1.1B* by Altering OsNLP4 Cytoplasmic-Nucleus Shuttling

**Fig. 4:**
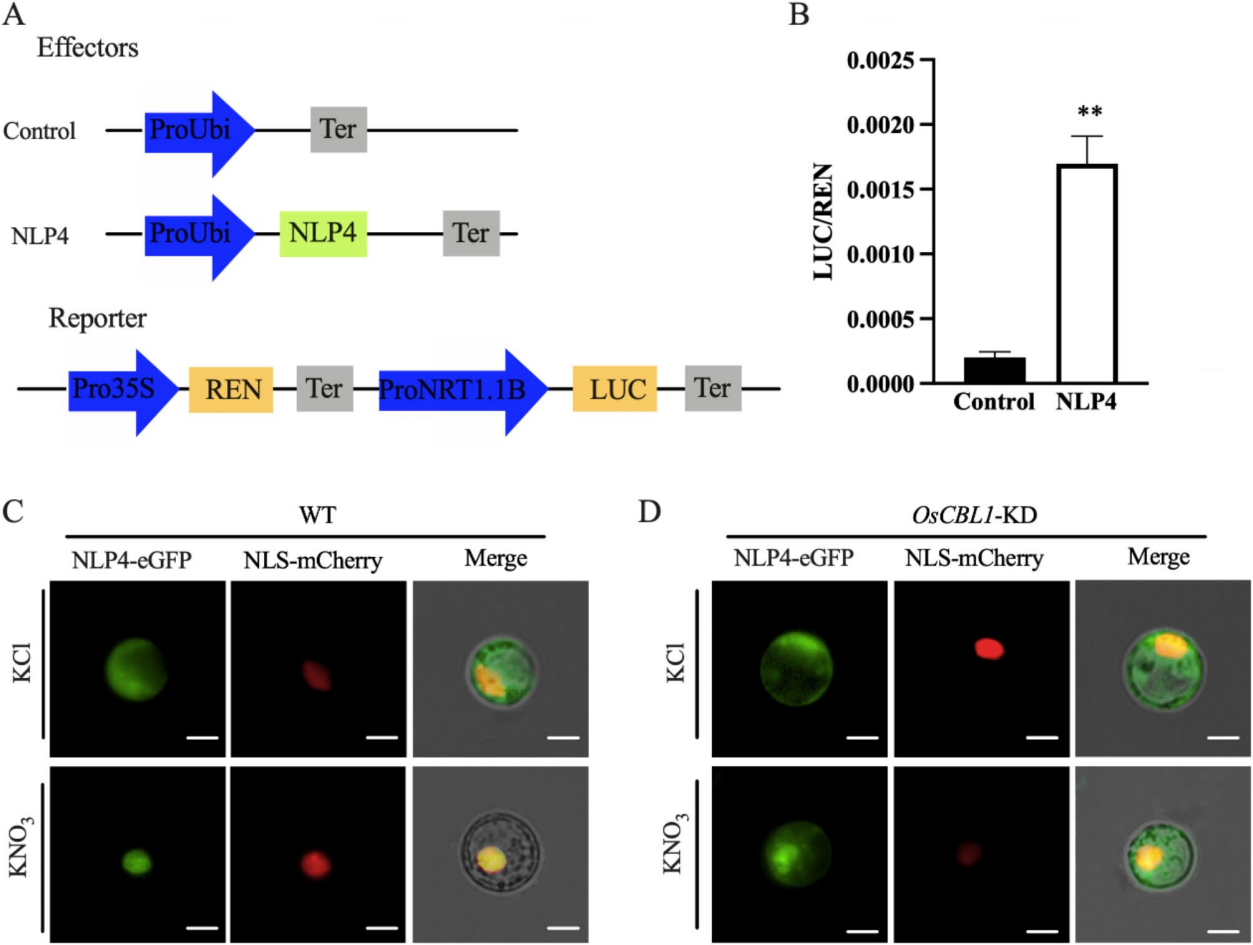
Knockdown of *OsCBL1* inhibits the nucleus aggregation of OsNLP4. **A** Schematic illustration of the effector and reporter constructs. **B** Dual-luciferase reporter analyze the transcriptional regulation of *OsNRT1.1B* promoter by OsNLP4 in rice protoplasts. *n* = 3 biologically independent samples. The error bars represent ± SEM. ** *P* < 0.01 compared to the control (*t*-test). **C, D** The impact of OsNLP4 subcellular localization in rice protoplasts of WT (**C**) and *OsCBL1*-KD (**D**) following nitrate (10 mM KNO_3_) induction. KCl treatment was used as the negative control. Scale bars, 5 μm. All experiments were repeated three times, and similar results were obtained

To elucidate the mechanism underlying the downregulation of *OsNRT1.1B* in *OsCBL1*-KD plants, we conducted an investigation into the transcription factor responsible for regulating the expression of *OsNRT1.1B.* Previous studies have indicated that OsNLP4, a member of the NIN-LIKE PROTEIN family (NLPs), plays a pivotal role in N signaling and assimilation. Moreover, OsNLP4 has been demonstrated to directly bind to the *OsNRT1.1B* promoter and *OsNRT1.1B* expression was downregulated in *osnlp4* mutants (Wu et al. 2021). Therefore, OsNLP4 attracted our interest for further research. A reporter gene fLUC controlled by the *OsNRT1.1B* promoter was co-transfected into protoplasts with an effector plasmid for OsNLP4 expression (Fig. 4A). As shown in Fig. 4B, co-expression of OsNLP4 increased *OsNRT1.1B* promoter activity, confirming the Wu et al. (2021) finding that OsNLP4 positively regulates *OsNRT1.1B* expression.

Although the expression of *OsNRT1.1B* reduce in *OsCBL1*-KD plants. However, no significant difference in the transcription level of *OsNLP4* was observed between *OsCBL1*-KD and WT plants (Fig. S6). Transcription factors are predominantly localized in the nucleus, where they regulate downstream genes expression. Previous studies have demonstrated that nitrate availability can facilitate the nuclear translocation of OsNLP4 in rice (Wu et al. 2021). Similarly, in *Arabidopsis*, the movement of AtNLP7 into the nucleus in response to nitrate depends on a Ca^2+^-sensor protein kinase (Liu et al. 2017). These observations imply that the Ca^2+^ sensor protein OsCBL1 may also be essential for the cytoplasmic-nucleus transport of OsNLP4. Therefore, we conducted an assay to determine the subcellular localization of OsNLP4 in both WT and *OsCBL1*-KD protoplasts under nitrate supply condition. The results showed no difference in the localization of OsNLP4-eGFP in WT and *OsCBL1*-KD rice protoplasts under KCl treatment. However, when exposed to nitrate, OsNLP4-eGFP was exclusively located in the nucleus of WT protoplasts, whereas it was present in both the cytoplasm and the nucleus of *OsCBL1*-KD protoplasts (Fig. 4C and D). This suggested nitrate-induced shift of OsNLP4 from the cytoplasm to the nucleus is inhibited in *OsCBL1*-KD plants. Considering the result of OsNLP4 increasing the expression of *OsNRT1.1B* in the LUC assay (Fig. 4B), we hypothesized that OsCBL1 may regulate the expression of *OsNRT1.1B* by controlling the nitrate-induced cytoplasmic-nuclear shuttling of OsNLP4.

## Discussion

Nitrate and phosphate serve dual roles as essential nutrients in plants, providing critical sources of nitrogen (N) and phosphorus (P) across all stages of growth, while also acting as signaling molecules that regulate gene expression and elicit nutritional responses. Previous studies have demonstrated that the Ca^2+^ sensor protein *OsCBL1* is involved in nitrate signaling, and *OsCBL1* knockdown leads to altered expression of genes responsive to both nitrate and phosphate (Yang et al. 2019; Hu et al. 2021). These findings suggest that *OsCBL1* functions as an integrator, mediating the crosstalk between nitrate and phosphate signaling pathways. To further elucidate the underlying molecular mechanisms, we conducted a comparative study of growth characteristics between *OsCBL1*-KD and WT plants under varying N and P supply conditions. Our results demonstrate that *OsCBL1*-KD plants exposed to high phosphate levels in the presence of elevated nitrate did not exhibit a significant increase in biomass compared to WT plants under the same conditions (Fig. 1). Moreover, the expression of nitrate-induced phosphate starvation-inducible (PSI) genes was reduced in *OsCBL1*-KD plants following short-term nitrate treatment, relative to WT plants (Fig. 1). These findings highlight the critical role of *OsCBL1* in coordinating N and P signaling. The downregulation of *OsCBL1* appears to impair the plant’s capacity to respond to nitrate-induced phosphate signaling, ultimately hindering biomass accumulation in the context of elevated nitrate and phosphate availability. These insights contribute to a deeper understanding of the regulatory mechanisms governing nitrate-induced phosphate signaling pathways.

The coordinated utilization of N and P is essential for achieving sustainable high crop yields. Hu et al. found that under low nitrate conditions in rice, the cytoplasmic repressor protein OsSPX4 binds to the transcription factors OsNLP3 and OsPHR2, preventing their nuclear translocation and inhibiting the expression of N and P response genes (Hu et al. 2019). Under elevated nitrate conditions, the nitrate transporter OsNRT1.1B interacts with OsSPX4 and the ubiquitin ligase OsNBIP1 to form the OsNRT1.1B-OsSPX4-OsNBIP1 complex. This complex mediates the ubiquitination and subsequent degradation of OsSPX4, thereby allowing OsNLP3 and OsPHR2 to translocate into the nucleus, where they activate the transcription of genes involved in N and P responses. This regulatory mechanism promotes balanced nutrient ratios and enhances nutrient use efficiency (Hu et al. 2019). In our experiments, the downregulation of PSI genes was observed in *OsCBL1*-KD plants under nitrate-deficient conditions (Fig. 1). Additionally, nitrate-induced degradation of OsSPX4 was significantly impaired in *OsCBL1*-KD protoplasts (Fig. 2). Furthermore, the transcriptional activity of *OsNRT1.1B* was markedly reduced in *OsCBL1*-KD plants across various conditions (Fig. 3), leading to diminished OsSPX4 degradation. These findings suggest that *OsCBL1* plays a key role in integrating N-P signaling by potentially modulating the OsNRT1.1B-OsSPX4 module in rice, thereby disrupting the balance between N and P. This observation provides valuable insights into the potential upstream regulators of the OsNRT1.1B-OsSPX4 module in maintaining N-P nutrient balance.

**Fig. 5:**
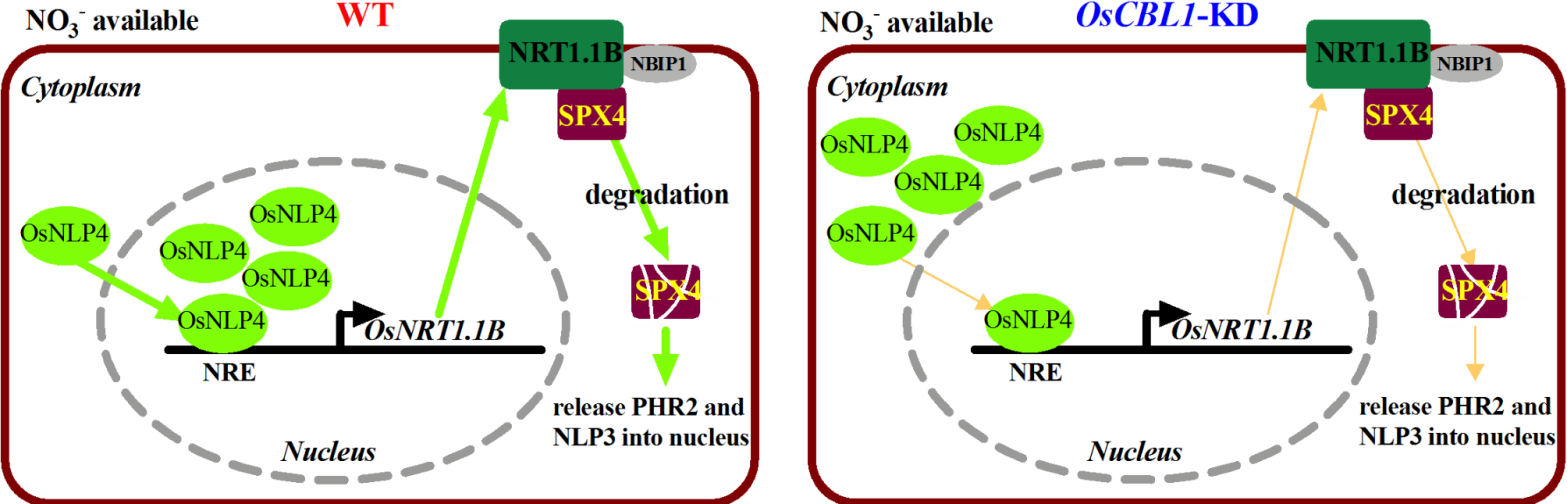
A proposed model of OsCBL1 modulates the nitrate-induced phosphate response. In the presence of nitrate, *OsCBL1* facilitates the nuclear translocation of OsNLP4 and its binding to NRE elements. This initiates the activation of *OsNRT1.1B* expression, leading to the degradation of OsSPX4. As a consequence, OsPHR2 and OsNLP3 are released, coordinating the stimulate downstream genes responses associated with phosphate and nitrate responses, respectively. The thickness of the lines represents the strength of control over the downstream genes

The calcium sensor protein OsCBL1 does not directly regulate the expression of *OsNRT1.1B*. Previous studies have shown that the expression of *NRT* genes can be modulated by NLP transcription factors, which bind to the NRE element within the gene promoter (Konishi and Yanagisawa 2013, 2014). Specifically, OsNLP1, OsNLP3, and OsNLP4 have been demonstrated to directly interact with the NRE element in the promoter of *OsNRT1.1B*. Moreover, it has been observed that *OsNLP3* and *OsNLP4* undergo nitrate-induced translocation from the cytoplasm to the nucleus, whereas OsNLP1 remains constitutively localized in the nucleus (Alfatih et al. 2020; Wu et al. 2021; Zhang et al. 2022). Hu et al. demonstrated that the interaction between OsSPX4 and OsNLP3 inhibits the nuclear translocation of OsNLP3 (Hu et al. 2019). Our study found that nitrate-induced degradation of OsSPX4 was absent in *OsCBL1*-KD plants, which may result in the retention of OsNLP3 in the cytoplasm through its interaction with OsSPX4. The expression levels of *OsNLP1* and *OsNLP4* remain unchanged in both WT and *OsCBL1*-KD plants (Fig. S6, Fig. S7). Given that OsNLP1 is constitutively localized in the nucleus, we performed a subcellular localization analysis of OsNLP4. The results showed that the translocation of OsNLP4 from the cytosol to the nucleus in response to nitrate was impaired in *OsCBL1*-KD plants (Fig. 4). This suggests that OsCBL1 regulates *OsNRT1.1B* expression by modulating the intracellular movement of OsNLP4 under high nitrate conditions, thereby stabilizing OsSPX4 and preventing the nuclear translocation of OsNLP3 and OsPHR2. As a result, the expression of N- and P-related genes is ultimately reduced (Fig. 5). However, further research is required to elucidate the mechanism by which OsCBL1 controls the cytoplasm-to-nucleus translocation of OsNLP4.

Previous studies have demonstrated that the OsNRT1.1B-OsSPX4 module is involved in nitrate-triggered phosphate signaling and the phosphate-mediated nitrate response (Hu et al. 2019). Given the role of *OsCBL1* in nitrate-triggered phosphate signaling, it is plausible that *OsCBL1* also contributes to regulating the nitrate response via phosphate. Notably, in WT plants, elevated phosphate levels have been shown to upregulate the expression of nitrate-response genes (*OsNRT2.1*, *OsNIR1*, and *OsNIA1*) under low nitrate conditions, while downregulating their expression under high nitrate conditions (Fig. S8). This suggests that the regulation of nitrate-response genes by phosphate availability is dependent on the surrounding nitrate concentration. Under low nitrate conditions, the application of high phosphate led to a significant upregulation of *OsNRT2.1*, *OsNIR1*, and *OsNIA1* expression in WT plants, with fold increases of 16, 4.5, and 7.7, respectively. In contrast, *OsCBL1*-KD plants exhibited a much weaker response to high phosphate, with fold increases ranging from 1.8 to 2.5 for *OsNRT2.1*, 1.5 to 1.7 for *OsNIR1*, and 0.7 to 1 for *OsNIA1* (Fig. S9A). Under high nitrate conditions, HP significantly reduced the expression of *OsNRT2.1*, *OsNIR1*, and *OsNIA1* in WT plants by factors of 1.9, 2.8, and 25, respectively. Conversely, in *OsCBL1*-KD plants under HNHP conditions, the reduction in expression of these genes was less pronounced, with fold decreases ranging from 0.12 to 0.26 for *OsNRT2.1*, 0.19 to 0.3 for *OsNIR1*, and 1 to 22 for *OsNIA1* (Fig. S9B). Additionally, nitrate content in the roots of WT plants increased under LNHP (compared to LNLP) but decreased under HNHP conditions (compared to HNLP) (Fig. S10). In contrast, the downregulation of *OsCBL1* impaired the phosphate-induced increase in nitrate content under LN conditions. Similarly, *OsCBL1* knockdown disrupted the increase in nitrate content under low phosphate conditions (Fig. S11). These results indicate that *OsCBL1* plays a critical role in mediating N-P interactions, warranting further investigation to fully understand its regulatory mechanisms.

The downregulation of *OsNRT1.1B* expression in *OsCBL1*-KD plants would be expected to reduce nitrate content, as *OsNRT1.1B* functions as a nitrate transporter. However, contrary to this expectation, *OsCBL1* knockdown actually resulted in increased nitrate content under various nitrate and phosphate conditions (Fig. S10). In our previous research, we discovered that the downregulation of *OsCBL1* leads to the upregulation of the nitrate transporter gene *OsNRT2.2* by suppressing the expression of OsCCA1, thereby increasing nitrate content in *OsCBL1*-KD plants (Hu et al. 2023). Accordingly, we analyzed *OsNRT2.2* expression in both *OsCBL1*-KD and WT plants under various nitrate and phosphate conditions, and observed a marked increase in *OsNRT2.2* expression in *OsCBL1*-KD plants compared to WT (Fig. S12). These findings suggest that *OsCBL1* regulates the expression of multiple nitrate transporter genes, and that fine-tuning the expression of these genes is crucial for nitrogen accumulation in rice. Notably, despite the higher nitrate content observed in *OsCBL1*-KD plants, the absence of OsSPX4 degradation, weaker induction of PSI genes, and lack of growth promotion by high phosphate in *OsCBL1*-KD plants were consistent with phenotypes observed in *osnrt1.1b* mutants (Hu et al. 2019). We propose that *OsCBL1* regulates *OsNRT1.1B* expression by modulating the movement of OsNLP4 in response to nitrate-induced phosphate signaling. Additionally, *OsCBL1* independently regulates *OsNRT2.2* expression to facilitate nitrate accumulation, suggesting that these are two distinct pathways.

## Conclusion

This study demonstrates that knockdown of *OsCBL1* disrupts the cytoplasmic-nuclear shuttle of OsNLP4 in response to nitrate, leading to reduced expression of *OsNRT1.1B* and subsequently impacting the nitrate-induced phosphate response through the stabilization of OsSPX4. This finding provides a novel clue to discover the upstream regulators of OsNRT1.1B-OsSPX4 module in maintaining N-P nutrient balance in rice, highlighting the potential significance of OsCBL1 as a crucial gene for optimizing the utilization of nitrogen and phosphorus nutrients in rice cultivation.

## Electronic Supplementary Material

Below is the link to the electronic supplementary material.

Supplementary Material 1

## Data Availability

No datasets were generated or analysed during the current study.

## Abbreviations

Pi: Phosphate
OsCBL1: Calcineurin B-like protein-1
NRE: Nitrate responsive cis-element
N: Nitrogen
P: Phosphorus
NLP: NIN-LIKE PROTEIN
PSI: Phosphate starvation-induced
REN: Renilla luciferase
fLUC: Firefly-luciferase
LN: Low nitrate
HN: High-nitrate
