# Supplementary material for: OsCBL1 Modulates the Nitrate-Induced Phosphate Response by Altering OsNLP4 Cytoplasmic-Nucleus Shuttling
Source: Rice (N Y). 2025 Mar 10;18:11. doi: 10.1186/s12284-025-00768-6 (PMC11891122; doi:10.1186/s12284-025-00768-6)
Supplement: Supplementary file 1 — Supplementary Material 1 [file 12284_2025_768_MOESM1_ESM.docx]

**Supplementary Information**

**
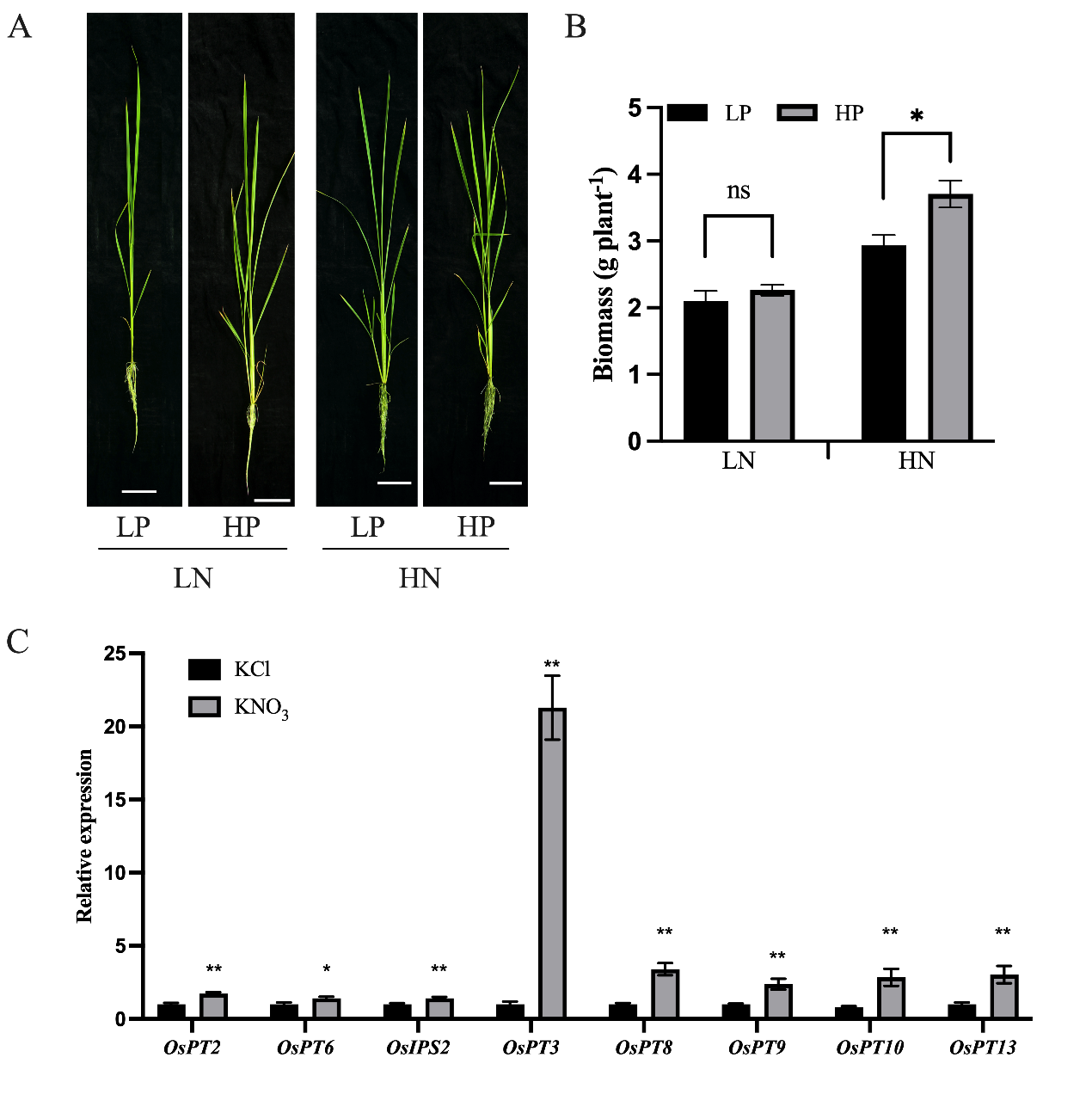
**

**Fig. S1** Phosphate utilization is induced by nitrate.

**A** The phenotype of WT plants under varying N and P conditions. High nitrate (HN), 5 mM KNO_3_; low nitrate (LN), 0.2 mM KNO_3_; high phosphate (HP), 0.18 mM KH_2_PO_4_; low phosphate (LP), 0.018 mM KH_2_PO_4_. Scale bars = 5 cm. Images are representative of 9 rice plants. **B** The biomass of WT plants under varying N and P conditions. n = 9 biologically independent samples. The error bars represent ± SEM. **P*< 0.05 compared to the LP condition (*t*-test). **C** The expression of PSI genes in WT plants following treatment with KNO_3_ (5 mM)or KCl (5 mM). n = 3 biologically independent samples. The error bars represent ± SEM. **P*< 0.05, and ***P*< 0.01 compared to the KCl(*t*-test).


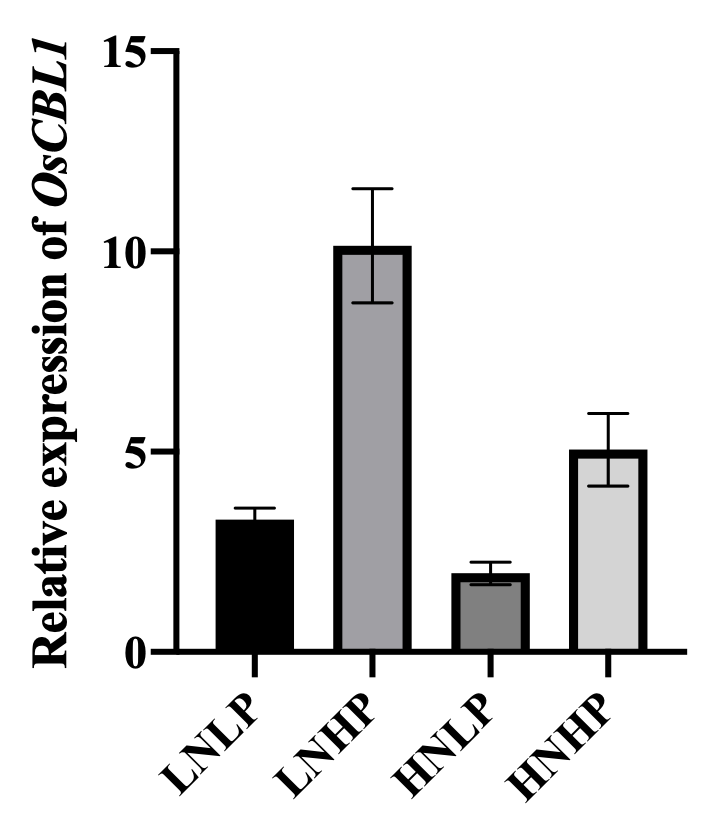


**Fig. S2** The expression pattern of *OsCBL1* in WT under different N and P conditions. n = 3 biologically independent samples. The error bars represent ± SEM.


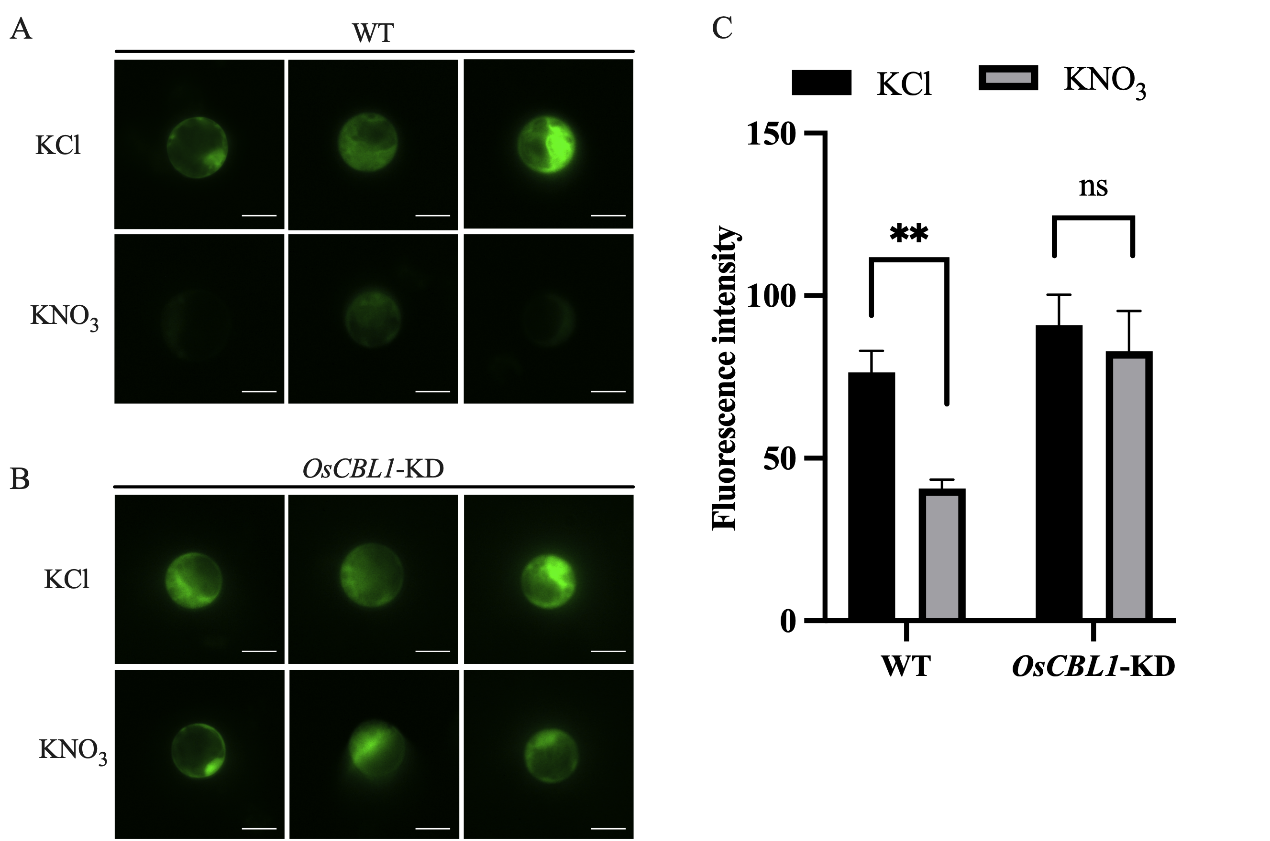


**Fig. S3**The fluorescence of OsSPX4-eGFP in rice protoplasts of WT (**A**) and *OsCBL1*-KD (**B**) plants following treatment with KNO_3_ (10 mM). KCl treatment was used as the negative control. Scale bars, 5 μm. The fluorescence intensity (**C**) was measured by Image J. All experiments were repeated three times, and similar results were obtained. The error bars represent ± SEM. ***P*< 0.01 compared to the KCl (*t*-test).


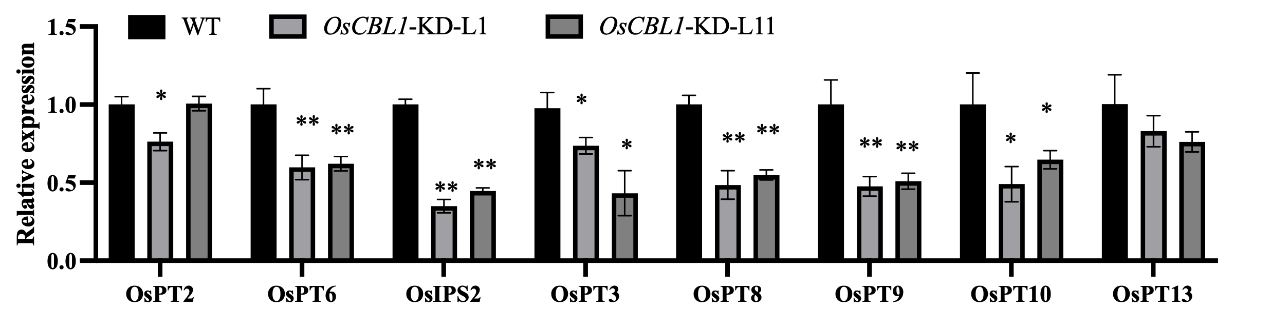


**Fig. S4**The expression of PSI genes in WT and *OsCBL1*-KD plants following treatment with nitrate induction. n = 3 biologically independent samples. The error bars represent ± SEM. **P*< 0.05, ***P*< 0.01 compared to the WT (*t*-test).


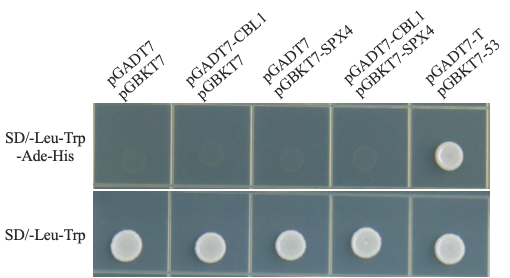


**Fig. S5**Yeast two-hybrid analysis of the interaction between OsCBL1 and OsSPX4.


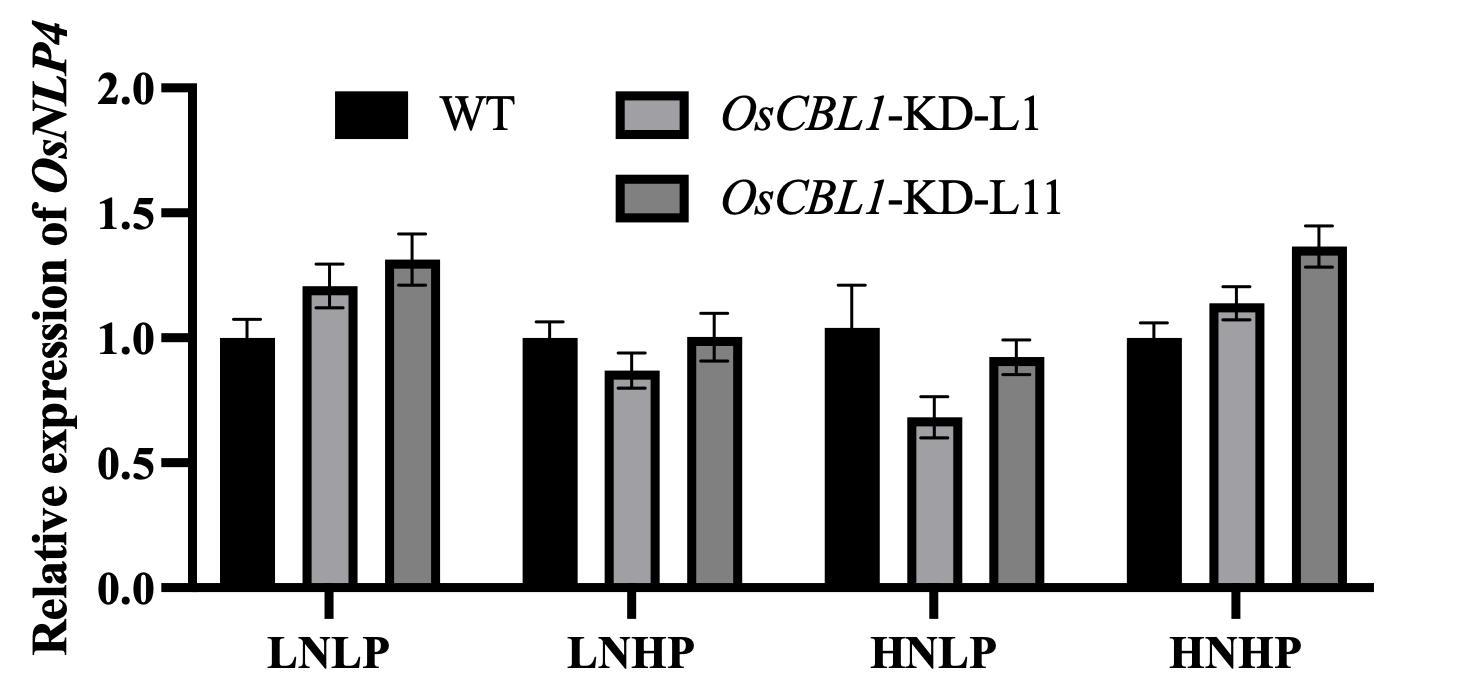


**Fig. S6**The expression of *OsNLP4* in WT and *OsCBL1-*KD plants under varying N and P conditions. n = 3 biologically independent samples. The error bars represent ± SEM.


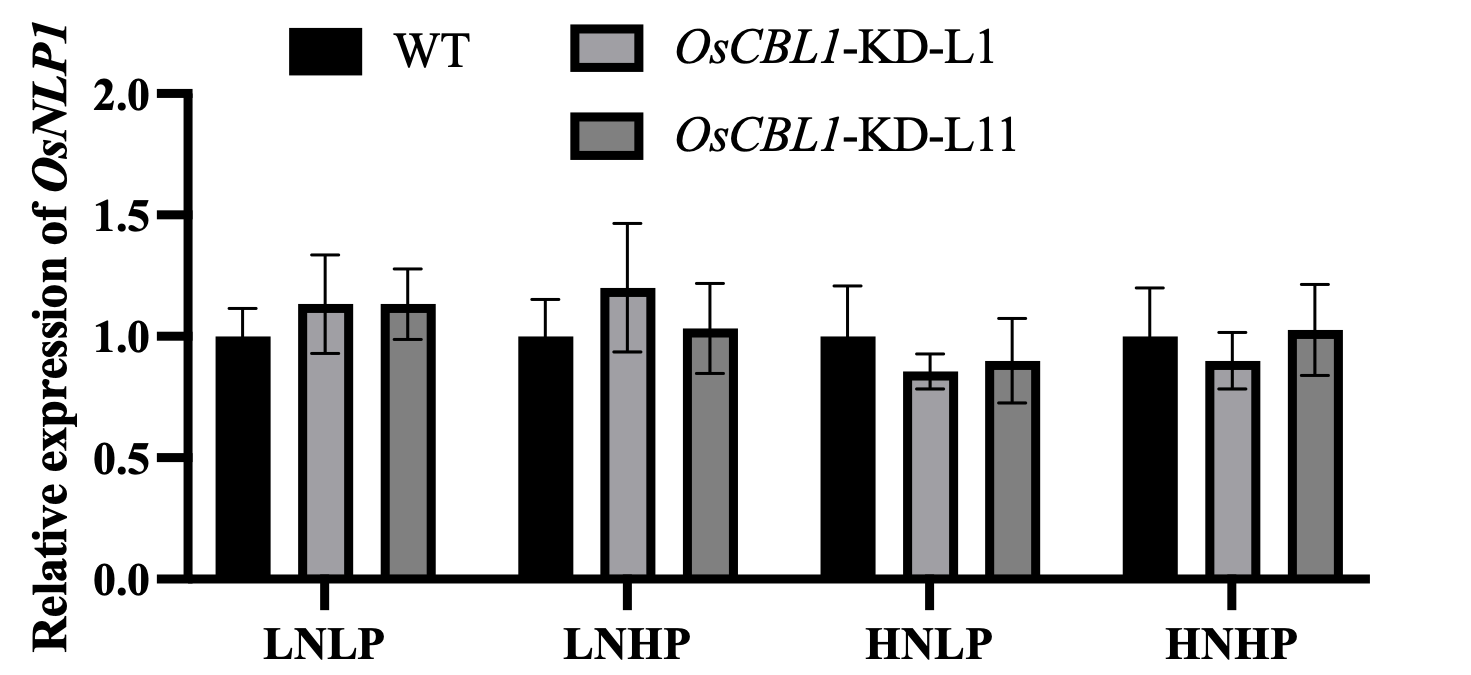


**Fig. S7**The expression of *OsNLP1* in WT and *OsCBL1-*KD plants under varying N and P conditions. n = 3 biologically independent samples. The error bars represent ± SEM.


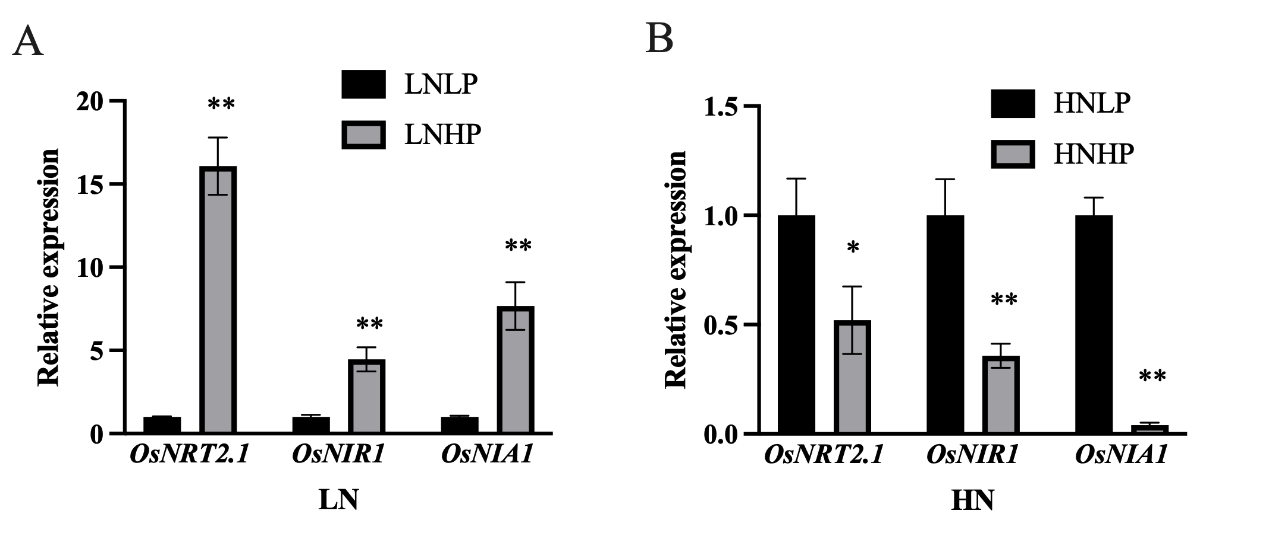


**Fig. S8**The expression of nitrate response genes in WT between different P supply under HN or LN conditions.

**A** The expression of *OsNRT2.1*, *OsNIR1* and *OsNIA1* in the roots of WT plants between different P supply under LN conditions. n = 3 biologically independent samples. The error bars represent ± SEM. ***P*< 0.01 compared to the WT (*t*-test). **B** The expression of *OsNRT2.1*, *OsNIR1* and *OsNIA1* in the roots of WT plants between different P supply under HN conditions. n = 3 biologically independent samples. The error bars represent ± SEM. **P*< 0.05, and ***P*< 0.01 compared to the WT (*t*-test).

**Fig. S9**The expression of nitrate response genes in WT and *OsCBL1*-KD plants between different P supply under HN or LN conditions.

**A** The induction assays of the expression of *OsNRT2.1*, *OsNIR1* and *OsNIA1* in the roots of WT and *OsCBL1*-KD plants between different P supply under LN conditions. n = 3 biologically independent samples. The error bars represent ± SEM. ***P*< 0.01 compared to the WT (*t*-test). **B** The induction assays of the expression of *OsNRT2.1*, *OsNIR1* and *OsNIA1* in the roots of WT and *OsCBL1*-KD plants between different P supply under HN conditions. n = 3 biologically independent samples. The error bars represent ± SEM. **P*< 0.05, and ***P*< 0.01 compared to the WT (*t*-test).


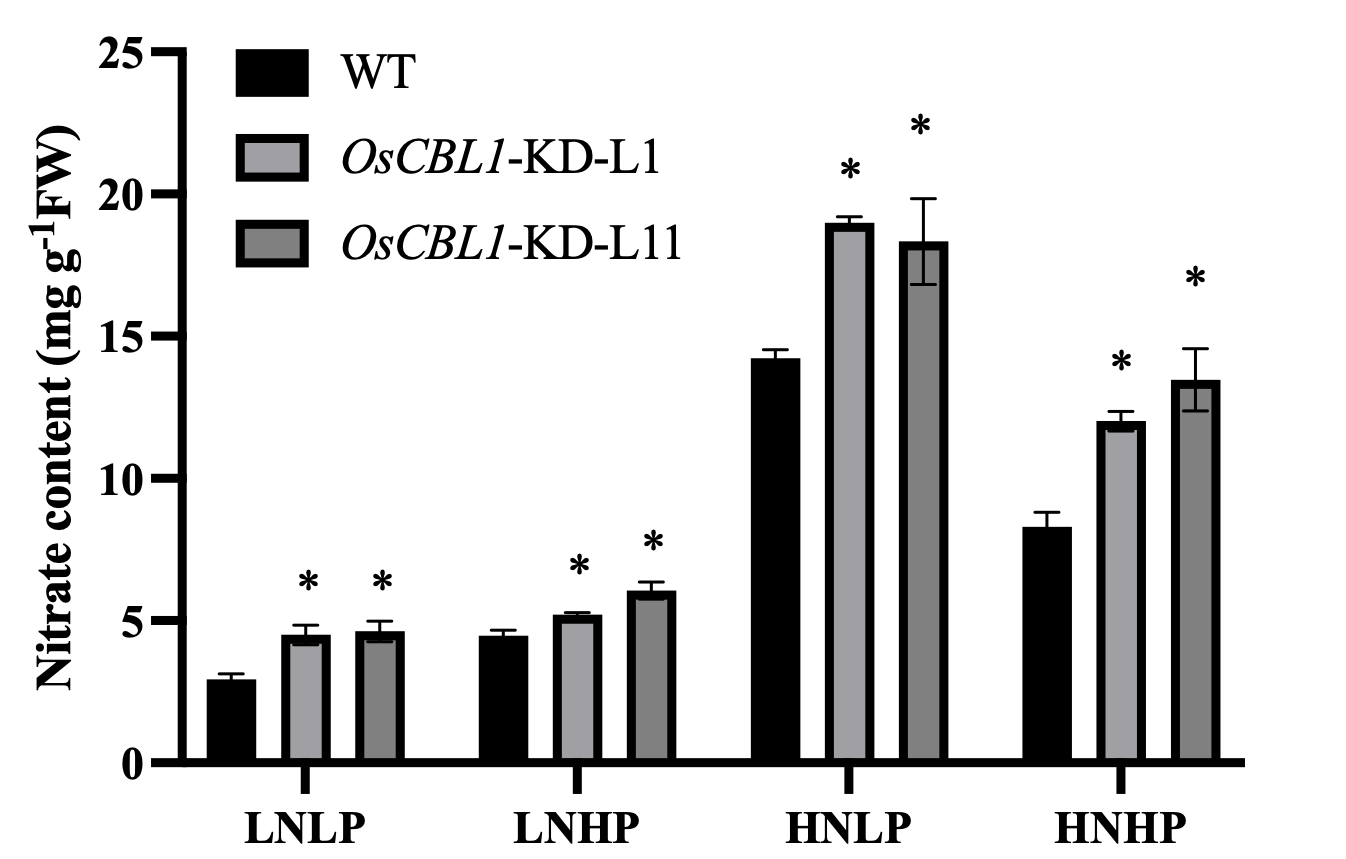


**Fig. S10** The nitrate content of *OsCBL1*-KD and WT under different nitrate and phosphate conditions. n = 3 biologically independent samples. The error bars represent ± SEM. **P*< 0.05 compared to the WT (*t*-test).


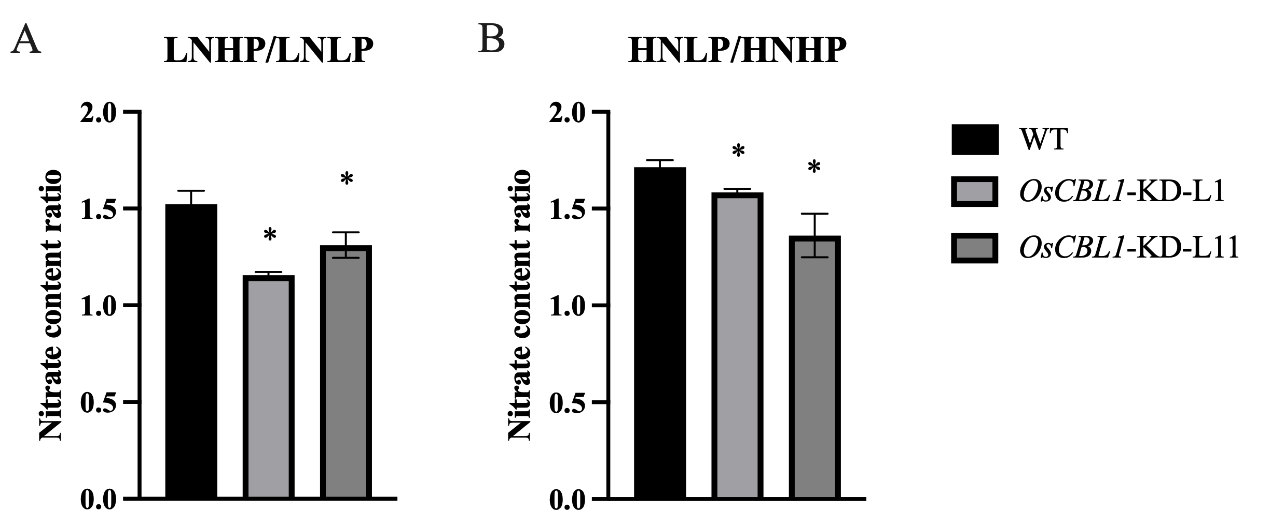


**Fig. S11**The nitrate content ratio of WT and *OsCBL1*-KD plants between different P supply under HN or LN conditions.

**A** The nitrate content ratio of WT and *OsCBL1*-KD plants under LN conditions. n = 3 biologically independent samples. The error bars represent ± SEM. **P*< 0.05 compared to the WT (*t*-test). **B** The nitrate content ratio of WT and *OsCBL1*-KD plants under HN conditions. n = 3 biologically independent samples. The error bars represent ± SEM. **P*< 0.05compared to the WT (*t*-test)


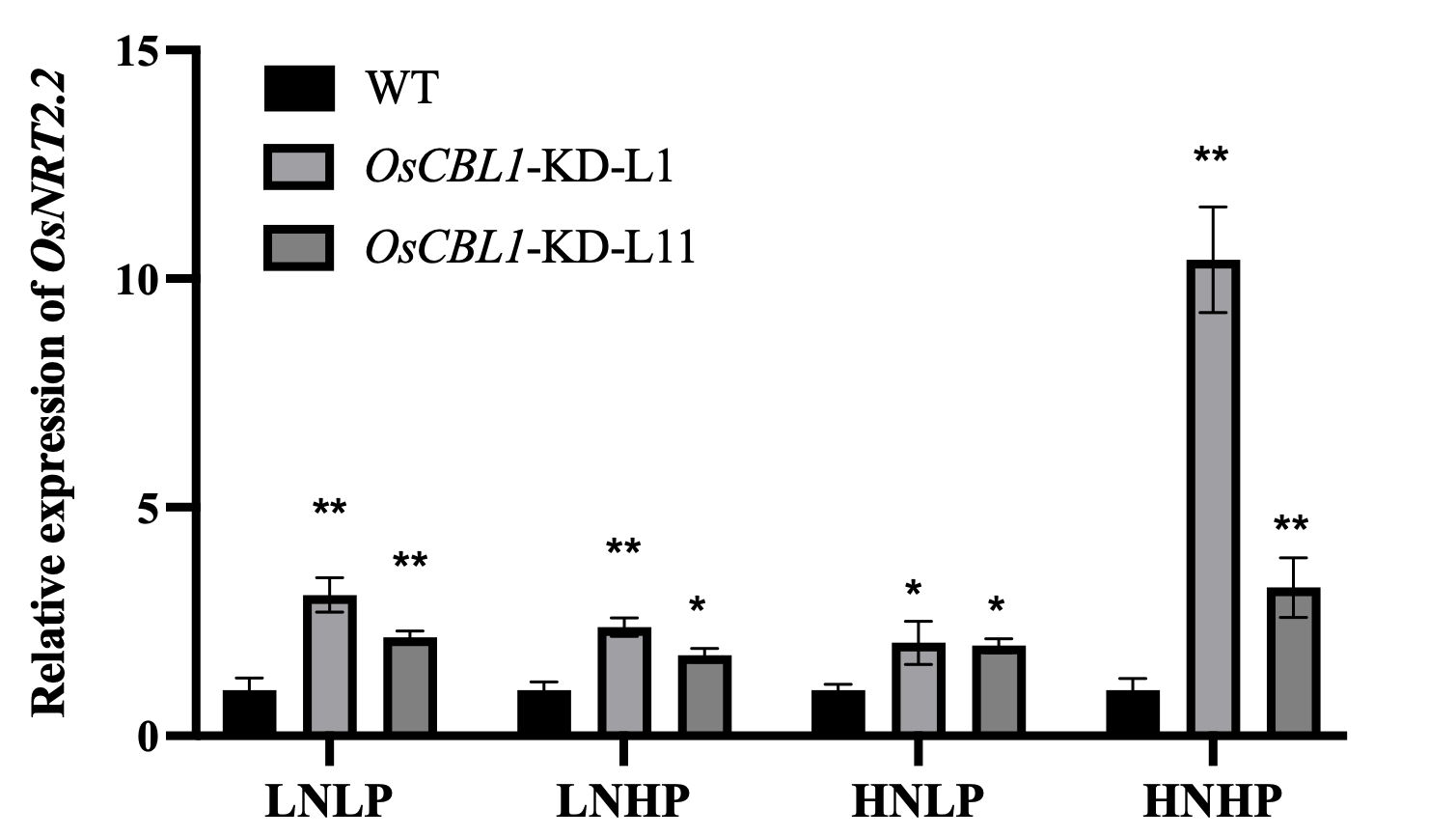


**Fig. S12** The expression of *OsNRT2.2* in *OsCBL1*-KD and WT under different nitrate and phosphate conditions. n = 3 biologically independent samples. The error bars represent ± SEM. **P*< 0.05, and ***P*< 0.01 compared to the WT (*t*-test).

**Table S1:** The primers used in this article.

| Primers for qPCR | |
| --- | --- |
| Primer name | sequence (5’-3’) |
| qOsACTIN1-F | ACCATTGGTGCTGAGCGTTT |
| qOsACTIN1-R | CGCAGCTTCCATTCCTATGAA |
| qOsPT2-F | CACAAACTTCCTCGGTATGCT |
| qOsPT2-R | GAAACCCCACAAATCCACAAC |
| qOsPT3-F | CGGGTACAAGCCAGGCATTG |
| qOsPT3-R | GCGACGTTCTCCTTGGACAC |
| qOsPT6-F | GCCCCTGCAAACTGTACTG |
| qOsPT6-R | AGCCAGGCCAGTTATATATCAAC |
| qOsPT8-F | CCTACTTGTGTTTGTCTATGTG |
| qOsPT8-R | GTGCCAAATTGCTGGTCTG |
| qOsPT9-F | GCATGATGTACGCGCTGTTC |
| qOsPT9-R | CTCTCGTTCTCCTCCAGCGA |
| qOsPT10-F | ATGTCGCCCATCCTTCCA |
| qOsPT10-R | TCGCTTTCCGACGATGATC |
| qOsPT13-F | TTAGCATAATTCTTTTAGTAGTTAAATAGGAGATG |
| qOsPT13-R | TGATTTAAGATAAGGATTGAATGCACAT |
| qOsIPS2-F | CCTTCTTCTGGATTCCTCTC |
| qOsIPS2-R | AGTTCACCACAAAAGATACAGTAG |
| qOsNRT1.1B-F | GGCAGGCTCGACTACTTCTA |
| qOsNRT1.1B-R | AGGCGCTTCTCCTTGTAGAC |
| qOsNLP4-F | TCCGATTCAAGCCCTTCCTG |
| qOsNLP4-R | TGTGCTGTTCACCATTTGCC |
| qOsNLP1-F | ACCGTTGCTCAATGCTTCCT |
| qOsNLP1-R | TACAACCAAAGGCAGCGTCA |
| Primers for transient expression assay | |
| Primer name | sequence (5’-3’) |
| pGreenII0800-Ubi-SPX4-LUC-SacII-F | ctagagcggccgccaccgcggATGAAATTCGGGAAGGATTTCA |
| pGreenII0800-Ubi-SPX4-LUC-SacII-R | ggaattcgatctccaccgcggTTCATCACGTGGCTGGCC |
| HBT-SPX4-eGFP-BamHI-F | ctccccttgctccgtggatccATGAAATTCGGGAAGGATTTCA |
| HBT-SPX4-eGFP-BamHI-R | gcccttgctcaccatggatccTTCATCACGTGGCTGGCC |
| pCAMBIA2300-NRT1.1B-BamHI-F | atctctggtacccggggatccATGGCGATGGTGTTGCCG |
| pCAMBIA2300-NRT1.1B-BamHI-R | caggtcgactctagaggatccTTAGTGGCCGACGGCGAT |
| pCAMBIA1301-UBI-NLP4-BamHI-F | caggtcgactctagaggatccATGGAAGAGGGAGACCCCCA |
| pCAMBIA1301-UBI-NLP4-BamHI-R | gatctgcaggtcgacggatccTCATGAGAAACCAGTGTGACCAA |
| pGreenII0800-NRT1.1B-promoter-BamHI-F | ttcctgcagcccgggggatccCATGATCTGCTCTGGTGTCTGTATT |
| pGreenII0800-NRT1.1Bpromoter-BamHI-R | cgctctagaactagtggatccCTTTCTATATCTAATTAATAACACTTTGGATTAG |
| HBT-NLP4-eGFP-BamHI-F | ctccccttgctccgtggatccATGGAAGAGGGAGACCCCCA |
| HBT-NLP4-eGFP-BamHI-R | gcccttgctcaccatggatccTGAGAAACCAGTGTGACCAA |
